# Clinical Utility of Mifepristone: Apprising the Expanding Horizons

**DOI:** 10.7759/cureus.28318

**Published:** 2022-08-23

**Authors:** Zalak V Karena, Harsh Shah, Hetvee Vaghela, Kalp Chauhan, Pranav K Desai, Asjad Raza Chitalwala

**Affiliations:** 1 Department of Obstetrics and Gynecology, Nootan Medical College and Research Centre, Visnagar, IND; 2 Department of Obstetrics and Gynecology, Pandit Deendayal Upadhyay Medical College, Rajkot, IND

**Keywords:** cushing disease, cushing syndrome, meningioma, leiomyoma, medical termination of pregnancy, abortion, mifepristone

## Abstract

Mifepristone is a progesterone and glucocorticoid receptor antagonist. Medical abortion with mifepristone and prostaglandin has revolutionized the abortion process extending abortion care to the doors of females. From as low as 2 mg/day to doses extending to 600 mg, from daily dosing to single dosage treatment, mifepristone has a wide perspective in the treatment of various pathologies. Cervical dilatation and myometrial contractility have made the utility of mifepristone feasible for second-trimester termination of pregnancy and induction of labor awaiting Food and Drug Administration approvals. Its anti-progesterone action on the menstrual cycle has a new dimension of use as a contraceptive, as well as use as a menstruation inductive agent. Its role in endometriosis, ectopic pregnancy, and adenomyosis requires more intensive research. Apoptotic action of mifepristone, interference of heterotypic cell adhesion to the basement membrane, cell migration, growth inhibition of various cancer cell lines, decreased epidermal growth factor expression, suppression of invasive and metastatic cancer potential, increase in tumor necrosis factor, downregulation of cyclin-dependent kinase 2, B-cell lymphoma 2, and Nuclear factor kappa B have opened its potential to be explored as anti-cancer treatment and its effects on leiomyoma. The drug needs to be studied more for the prospectus of its anti-glucocorticoid actions in a wider dimension beyond its acquiescence for the treatment of Cushing syndrome.

## Introduction and background

Mifepristone, a synthetic steroid, is an antagonist to glucocorticoid and progesterone receptors. At low doses, mifepristone binds to the intracellular progesterone receptor. At high doses, it blocks the glucocorticoid receptor affecting the hypothalamic-pituitary-adrenal axis and increasing circulating cortisol. Mifepristone has a higher affinity for the glucocorticoid II receptor than it does for the glucocorticoid I receptor [1].

Historically, the discovery of the compound RU 486, i.e., mifepristone is attributed to the search and investigation of a compound that would block glucocorticoid receptors. It was noticed that the compound was strongly bound to the identical progesterone receptors. This study further led to the evolution of the drug mifepristone and its introduction as an abortifacient due to its effect on the menstrual cycle and early pregnancy in 1982 [2]. A clinical study on mifepristone was conducted in 1984-1986. In France in 1988 and Britain in 1991, mifepristone was approved for clinical use in abortion. Later, in 1999, it was approved for clinical use in abortion in 10 European countries and Israel. Then, in 1992, mifepristone was approved for clinical use in China, and in 2000 in the United States [3,4] for abortion.

Slow metabolic clearance of mifepristone favors a single dose administration in the medical termination of pregnancy. The metabolic clearance rate of mifepristone is 30 L/day, and it has a half-life of 20 hours [5]. A low dose of mifepristone acts on the endometrial milieu but does not inhibit ovulation, and this action is attributed to its post-coital contraceptive action. High doses of up to 1,200 mg/day are used in Cushing syndrome. However, much higher doses in some cases have been linked to cholestatic liver disease.

Owing to its anti-progesterone and anti-glucocorticoid action, mifepristone has found wide clinical application. Not all applications are approved and require further high-grade randomized studies. Those with unsatisfactory results for a particular disease have opened a dimension of study for chemically similar drugs from the same chain. This review aims to discuss and focus on the possible wider utility of the drug which needs to be strengthened with better clinical evidence.

## Review

Methodology

An extensive review of the literature was done using PubMed, Web of Science, Google Scholar, and Scopus using the Medical Subject Headings (MeSH) term mifepristone. Studies and articles focusing on the clinical utility of mifepristone were included in this narrative review.

Obstetric and gynecological uses of mifepristone

Food and Drug Administration (FDA)-approved indications of mifepristone are early pregnancy termination combined with misoprostol and the treatment of hyperglycemia in Cushing syndrome [1]. In the following, we discuss the various uses of mifepristone that are approved and those that have potential application after future research [6].

Early Pregnancy Termination

Mifepristone decreases estrogen and progesterone receptors in decidua by acting as a competitive receptor antagonist of progesterone [7]. This is the probably attributable mechanism of action to the termination of pregnancy. Because mifepristone alone for the treatment of abortion has a success rate of 80%, small doses of uterotonic agents after mifepristone treatment is the preferred regimen in an attempt to increase complete abortion rates [8,9]. The lack of response could be due to an inadequate increase in either endogenous accumulation of prostaglandin F2α (PGF2α) or in uterine contractility which is subsequently overcome by the use of prostaglandins [10]. The FDA-approved regimen (2016) for early termination of pregnancy is mifepristone with misoprostol in pregnancy with 70 days or less since the first day of a woman’s last menstrual period. The approved mifepristone dosing regimen is 200 mg of mifepristone taken by mouth on day one, followed by 800 µg of misoprostol taken buccally after 24-48 hours. Follow-up with the healthcare provider is done about seven to fourteen days after taking mifepristone.

The mifepristone-misoprostol regimen in the late first trimester (>70 to ≤84-day gestation) is not FDA-approved compared with surgical abortion, which is effective and safer in the late first trimester (94.6% versus 97.9% complete abortion) and more effective than misoprostol alone (90.4 versus 81.6% complete abortion). Complete success rates are higher with repeat dosing of misoprostol [11].

Medical abortion with sequential administration of mifepristone and prostaglandin is a safe, effective, well-tolerated, and acceptable method of termination of early first-trimester pregnancy. The introduction of the mifepristone drug for abortion has introduced abortion care to the doorsteps of women. Especially in low and middle-income groups of countries where inpatient healthcare facilities are scarce in remote places and surgical treatment of medical termination of pregnancy may require more human resources and capacity building of healthcare logistics, mifepristone treatment, which is given as outpatient treatment for abortion, is revolutionary and a boon. The major side effect of this regimen is gastrointestinal and is commonly related to prostaglandin analog; however, these are self-limiting. The latent period to the end result of abortion after mifepristone ingestion has raised a new concern requiring the investigation of the safety of the drug for the fetus of the mother who is set back from subsequent misoprostol ingestion and alters the abortion decision. After the intake of mifepristone, if the woman intends to continue the pregnancy, expectant management and fetal surveillance are done. However, studies now suggest that continuing pregnancy is more common with lower mifepristone doses and advanced gestational age. About 67% of women after mifepristone ingestion continue their pregnancy to term [12].

Second-Trimester Termination of Pregnancy

Mifepristone has been studied for its use in second-trimester termination of pregnancy with promising results in combination with misoprostol and, less commonly, oxytocin. Second-trimester termination of pregnancy can be done both medically and surgically. Induced abortion with a mifepristone and misoprostol regimen is the preferred approach; where mifepristone is not available, misoprostol alone for medical abortion can also be effective over dilation and evacuation (D&E), contributing significantly to safety [13]. Mifepristone followed by an oxytocin regimen has a long time until expulsion but has fewer side effects compared to the mifepristone-misoprostol regime [14]. Administering mifepristone and misoprostol simultaneously over the sequential dosing protocol results in lower expulsion rates within 24 hours of taking misoprostol, longer median misoprostol treatment times, and requires higher misoprostol doses. Both regimens work equally effectively at 48 hours. Simultaneous dosing results in less total time from the first clinical contact to complete abortion. Other methods used in second-trimester abortion are a combination of prostaglandin and hygroscopic dilator combination, namely, sulprostone and Dilapan. Significantly shorter hospitalization stays and short treatment to abortion intervals in patients treated with mifepristone and misoprostol compared to sulprostone and Dilapan treatment have been observed [15].

Missed Abortion and Fetal Demise

Mifepristone is as effective as other conventional methods in the termination of pregnancy in case of fetal demise in all gestational ages. Treatment with mifepristone plus misoprostol has a higher success rate and is more cost-effective than misoprostol alone in the management of missed miscarriages [16-18]. It is used to induce labor after intrauterine fetal demise. Various studies have found mifepristone-misoprostol to be more effective than misoprostol alone in 14-28-week gestational fetal death [19,20]. Short induction to the delivery time and higher success rate have been noted with the mifepristone group compared to misoprostol alone with intrauterine fetal demise over 28 weeks of gestation [21].

Menstrual Induction

Menstrual induction refers to early uterine evacuation without laboratory confirmation of pregnancy in women with delayed menses usually up to 10 days after the expected date. Mifepristone may hold promise for “menstrual regulation” and induction in case of missed dates of the period for women who do not have access to medical confirmation of pregnancy and for women who do not want to determine pregnancy. Various doses (150 mg, followed by misoprostol, 600 mg single dose) have been studied with promising results [22-24].

Emergency Contraception

Progesterone receptor antagonists such as mifepristone, selective progesterone receptor modulators such as ulipristal, and selective estrogen receptor modulators such as centchroman are the newly emerging drugs in emergency contraception. The mechanism of action of mifepristone depends on the timing of the administration in the menstrual cycle [25]. After the dominant follicle is developed, during the follicular phase, mifepristone delays the rise in estrogen, luteinizing hormone (LH) surge, ovulation, and subsequent follicular and endometrial development. These effects of mifepristone ultimately lead to the inhibition of ovulation [25,26]. If taken after ovulation, mifepristone inhibits endometrial development and blocks the expression of necessary endometrial receptors. The endometrium remains immature, thus preventing implantation from effectively occurring [25-28]. Both low‐dose mifepristone (less than 25 mg) and mid‐dose mifepristone (25-50 mg) within 120 hours of unprotected intercourse are associated with fewer pregnancies than levonorgestrel and Yuzpe [29].

Estrogen-Free Contraceptive Pill

Given in the first three days of the menstrual cycle, mifepristone does not affect the cycle, length of the follicular phase, LH surge, or luteal phase duration. Daily doses between 2 and 5 mg inhibit ovulation and menstruation in over 90% of cycles while maintaining follicular development and estradiol levels within the range found during the follicular phase with the endometrium showing down-regulation of progesterone receptors (PR). The antiproliferative effect of mifepristone is reassuring suggesting that the risk of atypical hyperplasia due to the effect of prolonged exposure to estrogen unopposed by progesterone is low. This safety spectrum by mifepristone is an impetus for future studies [30-34]. Owing to the failure rate of 17-19%, in addition to low efficacy and its limitation such as the disruption of cycle rhythm, its use is not popular. However, higher doses are more effective and need further good-quality randomized studies to testify to its efficacy to recommend its clinical use. A 200 mg mifepristone tablet on the 16th day of the menstrual cycle as a monthly pill has been found to have a high success rate and better acceptance rate in educated females [35]. Once weekly administration with 25 mg of mifepristone is also a potentially effective method for regular contraception [36].

Cervical Dilatation

Mifepristone is useful for the preoperative preparation of women for surgical abortion in the late first-trimester and second-trimester pretreatment because of its marked effect on cervical dilatation and myometrial contractility. It reduces the interval between expulsion and prostaglandin administration. However, the findings regarding the use of mifepristone in preoperative hysteroscopy patients for cervical dilatation are not consistent and promising [37-39].

Induction of Labor

Mifepristone is efficient in inducing labor in term pregnancy. There are no significant adverse maternal and neonatal outcomes between mifepristone use and expectant management. However, more painful uterine contractions and a higher rate of cephalopelvic disproportion have been directly related to the mifepristone action in some studies requiring further verification [40,41]. A higher success rate with mifepristone and fewer chances of failure of induction with mifepristone that leads to cesarean section justify its future trial for use in induction of labor [42].

Ectopic Pregnancy

A recent meta-analysis of 23 randomized studies involving 1,706 patients concluded that a combination of mifepristone and methotrexate results in better outcomes of ectopic pregnancy [43]. A combination of methotrexate and mifepristone increases the success rate, especially if the progesterone level is higher [44,45]. However, this conclusion needs further verification by randomized, double-blind, and controlled trials with larger sample sizes and more rigorous trial designs.

Endometriosis

As an antiprogesterone, mifepristone causes upregulation of endometrial androgen receptors in both glandular and stromal cells, and this has an antiproliferative and antiestrogenic effect that inhibits estradiol-stimulated endometrial growth. This is the probable mechanism of action of mifepristone in endometriosis treatment. Studies conducted to date show some relief of symptoms with mifepristone. Pelvic pain is improved with a low dose of 5 mg mifepristone for three months. However, no change in the extent of the disease has been noted on follow-up laparoscopy at such low doses. A dose of 50 mg of mifepristone for six months demonstrates a significant regression in visible endometriotic lesions in follow-up laparoscopy and a decrease in clinical symptoms [46-48].

Leiomyoma

Mifepristone antagonizes the action of progesterone required for the maintenance and proliferation of leiomyomas [49]. Progesterone mediates the growth of fibroids by B-cell lymphoma 2 (BCL-2) induction [50]. Mifepristone reduces BCL-2, decreases epidermal growth factor (EGF) expression in fibroids, and increases tumor necrosis factor (TNF) and androgen receptors [51]. A size reduction may be due to its direct effect on PRs. Following mifepristone treatment in leiomyoma, amenorrhoea ensues because of ovulation inhibition. Repressing the effect on endometrial vasculature and stromal vascular endothelial growth factor decreases menstrual blood loss [52-55]. Mifepristone therapy with 25 mg daily is the recommended dosing. In a trial with a 25 mg dosage, there was a 49% reduction in tumor volume after three months in uterine leiomyoma [56]. Another study concluded that there is a 90% reduction in menstrual blood loss with both 10 and 25 mg of mifepristone for three months in fibroid patients. However, a 25 mg dose has a significantly greater reduction in the size of myoma than 10 mg with no endometrial atypia [55]. For patients who want to avoid surgery, at a young age, in premenopausal patients, as well as an adjunct to surgery for size reduction, mifepristone is a good choice. However, the notable adverse effect was the development of benign endometrial hyperplasia due to prolonged unopposed estrogen milieu [57].

Adenomyosis

Mifepristone causes cell cycle arrest by inhibiting cyclin-dependent kinase 1 (CDK1) and CDK2 expressions and induces cell apoptosis via the mitochondria-dependent signaling pathway and increased expression of caspase 3 in endometrial epithelial cells and stromal cells of adenomyosis. A decrease in C-X-C chemokine receptor type 4 (CXCR4) expression restricts the invasion of endometrial cells via the suppression of epithelial-mesenchymal transition. It restricts the migration of endometrial and stromal cells in adenomyosis. Therefore mifepristone can effectively inhibit the emergence and development of adenomyosis, decrease the uterine volume and cancer antigen-125 (CA-125) concentration, and increases the hemoglobin concentration in serum for adenomyosis patients [58,59]. In an interventional study of 20 adenomyosis patients on mifepristone 5 mg/day, there was significant relief concerning dysmenorrhea. The immunohistochemistry of these patients after treatment with mifepristone showed reduced secretion of interleukin-6 (IL-6) and TNF-α from endometrial epithelial and stromal cells, restricted infiltration, and degranulation of mast cells in eutopic and ectopic endometrium, as well as decreased density of nerve fibers by inhibiting the migration capacity of nerve cells in adenomyosis [60].

Breakthrough Bleeding in Levonorgestrel and Depot Medroxyprogesterone

In a study by John et al., a 50 mg dose of mifepristone administered every two weeks decreased the incidence of breakthrough bleeding (BTB) in new starters of depot medroxyprogesterone (DMPA). This effect may be attributed to the modulation of endometrial estrogen and progesterone receptors [61,62]. Mifepristone as pretreatment is effective in reducing the number of episodes and duration of intermenstrual bleeding/spotting in levonorgestrel-releasing intrauterine system (LNG-IUS) users, given at a dose of 100 mg every 30 days for three months. In a study with a 25 mg daily dosage of mifepristone, there was a significant reduction in bleeding and spotting days [63,64]. Hence, mifepristone use is promising in the prevention of BTB in DMPA and LNG users. However, the further scope of study in determining the optimum dose, schedule, and duration needs to be emphasized.

Premature Luteinizing Hormone Surges Undergoing Controlled Ovarian Hyperstimulation in In Vitro Fertilization

A single dose of mifepristone administered during the preovulatory phase of the cycle delays the LH surge and postpones ovulation [65]. Cycles with controlled ovarian hyperstimulation are associated with high early luteal progesterone levels and advanced endometrial histology. Low doses of mifepristone may correct precocious luteinization and restore endometrial receptivity. However, with further research, the dose of mifepristone needs to be standardized [66,67]. Various dosages (40 mg daily or 2.5 mg for two doses) and timing schedules (depending upon the COH protocol used, continuous administration from day two with follicle-stimulating hormone or after follicle aspiration and on the following day) for its efficacy needs to be studied ahead. Table 1 shows the obstetric and gynecological uses of mifepristone.

**Table 1:** Obstetric and gynecological uses of mifepristone. FDA: Food and Drug Administration; LNG: levonorgestrel; DMPA: depot medroxyprogesterone acetate; LH: luteinizing hormone; IVF: in vitro fertilization

Obstetric and gynecological uses	Indications	Recommended doses
FDA-approved use		
1	Early pregnancy termination	Mifepristone 200 mg orally on day 1, misoprostol 800 µg buccally after 24-48 hours
Proposed uses		
1	Second-trimester termination of pregnancy	Mifepristone 200 mg orally, followed by misoprostol 200 μg vaginally 3 hourly [15]
2	Missed abortion and fetal demise	600 mg/day for 2 days [19]; 200 mg orally with misoprostol (14-28 weeks of gestation) [20]
3	Menstrual induction	Mifepristone 600 mg single dose [22]; mifepristone 150 mg, misoprostol 0.4 mg vaginally after 2 days [24]
4	Emergency contraception	Less than 25 mg single dose [29]; 25-50 mg single dose [29]
5	Estrogen-free contraceptive pill	2 and 5 mg daily [30]; 25 mg once weekly [36]; 200 mg once a month on the 16th day of the menstrual cycle [35]
6	Cervical dilatation	600 mg orally single dose [39]
7	Induction of labor	200 mg single dose [42]
8	Ectopic pregnancy	Methotrexate 50 mg/m^2^ intramuscularly and mifepristone 600 mg orally [44,45]
9	Endometriosis	50 and 100 mg daily for 6 months [46]; 50 mg for 6 months [47]
10	Leiomyomas	10-25 mg daily for 3 months [55]
11	Adenomyosis	5 mg daily [60]
12	Breakthrough bleeding in LNG and DMPA	50 mg every 2 weeks for 24 weeks [61]; 100 mg every 30 days for 3 months [63]
13	Premature LH surges undergoing controlled ovarian hyperstimulation in IVF	2.5 mg two doses after follicle aspiration [66]; 40 mg daily [67]

Non-gynecological uses of mifepristone

Cushing Syndrome

**Figure 1:**
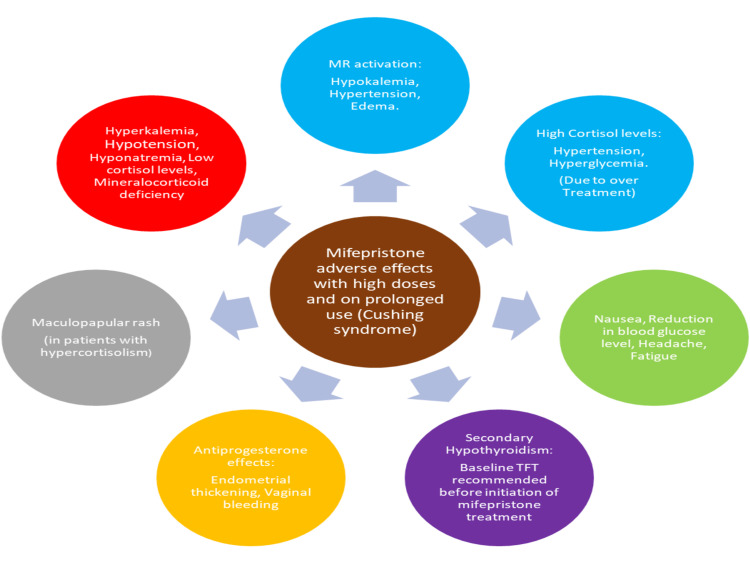
Adverse effects of mifepristone with high doses. MR: mineralocorticoid receptor; TFT: thyroid function test; blue circle: effects due to excess glucocorticoid receptor antagonism; red circle: effects due to adrenal insufficiency; green circle: effects due to cortisol withdrawal

Mifepristone alone is approved as a therapy for Cushing syndrome to control hyperglycemia in patients who are not candidates for surgical treatment or have not achieved remission from surgery for oral once-a-day administration [6,68-70]. Mifepristone is an effective treatment option in endogenous hypercortisolism, where monitoring and specific clinical considerations will benefit the patient. Mifepristone is started at an initial dose of 300 mg once a day orally, which can be increased up to 300 mg every fortnight to a maximum dose of 1,200 mg per day for the treatment of Cushing syndrome. Symptoms of cortisol withdrawal, hypokalemia, and change in thyroid function effects related to its antiprogesterone activity and rash require special consideration [71]. High-dose and prolonged use of mifepristone in Cushing syndrome brings about a concern about the adverse effects (Figure 1), which require good monitoring and patient care. In any condition that requires prolonged high-dose treatment of mifepristone, these adverse effects should be kept in mind and necessary precautions should be taken.

Insulin Resistance and Adipose Tissue Insulin Sensitivity

Short-term mifepristone administration improves adipose and hepatic insulin sensitivity in obese individuals with hyperglycemia without hypercortisolism [72]. It is a possible remedy to be studied for metabolic syndrome.

Breast Cancer, Ovarian Cancer, and Prostatic Cancer

Estrogen receptor (ER)-negative breast cancer with glucocorticoid receptor (GR) overexpression is associated with poor prognosis. GR antagonist with chemotherapy (nab-paclitaxel dose of 100 mg/m^2^ plus mifepristone 300 mg) helps to manage the toxicity of the former [73]. Mifepristone suppresses triple-negative breast cancer (TNBC) cell growth by inhibiting KLF5 expression via inducing miR-153. However, even at high doses, it has limited anticancer efficacy. To enhance the anticancer activities, a targeted compound chain containing 17 compounds is being designed and synthesized by altering the sensitive metabolic region of mifepristone [74]. Genetic deletion of the PR or its blockade by mifepristone-like progesterone antagonists effectively suppresses the development of high-grade squamous carcinoma (HGSC) of the ovary and its peritoneal metastases. Mifepristone treatment has been found to improve the survival rate in mice with HGSC. Hence, in women carrying a deleterious mutation in the *BRCA *gene, mifepristone can have a potential role [75,76]. Glucocorticoid and androgen receptor antagonist combination, mifepristone, and enzalutamide are safe and well tolerated for metastatic castration-resistant prostate cancer, but they do not result in PSA progression-free survival (PFS), with radiographic PFS, and PSA response rate (RR) earlier to monotherapy with enzalutamide. The development of more specific GR antagonists combined with AR antagonists, potentially studied in an earlier disease state, should be explored [77].

Meningioma

Owing to the antiprogestational activity of mifepristone, it is used as monotherapy for PR-positive benign central nervous system tumors, such as meningioma, at a dose of 200 mg/day. Usually, the long-term treatments are well tolerated; however, endometrial hyperplasia in some patients is noted and requires histological evaluation. Biochemical hypothyroidism is commonly associated with long-term mifepristone therapy in meningioma and requires periodic thyroid function screening [78-83].

Uveal Melanoma, Glaucoma, and Chorioretinopathy

In a study, Prisca et al. [84] reported that mifepristone impedes the proliferation of uveal melanoma cells in a concentration-dependent manner. More studies are required to determine the effects. Topical mifepristone preparation is being evaluated for the treatment of corticosteroid-induced intraocular pressure [85-87].

Psychiatric Diseases

Dysregulation of the hypothalamic-pituitary-adrenal axis and high cortisol levels have been implicated in mood, psychotic, and other psychiatric disorders. Mifepristone is a potent GR antagonist. Mifepristone blocks the cortisol feedback mechanism and raises circulating cortisol levels. Mifepristone blocks GR in key brain regions and monoaminergic nuclei, resulting in symptomatic and cognitive improvement. It has been found beneficial in the treatment of psychotic depression, cognitive dysfunction in schizophrenia and bipolar disorder, alcohol and cocaine dependence [88,89], posttraumatic stress disorder, weight gain associated with the use of antipsychotic drugs such as olanzapine [90-92], and Alzheimer’s disease [93] at doses of 600 mg to 1,800 mg per day. Table 2 describes the various non-gynecological uses of mifepristone.

**Table 2:** Non-gynecological uses of mifepristone. CS: Cushing syndrome; DM: diabetes mellitus; GR: glucocorticoid receptor; TNBC: triple-negative breast cancer; GBM: glioblastoma multiforme; IOP: intraocular pressure; HPA: hypothalamic-pituitary-adrenal; GABA: gamma-aminobutyric acid; AD: Alzheimer’s disease

Non-gynecological uses	Author	Type of study	Study details	Conclusions
Cushing syndrome	Morgan and Laufgraben [68]	Review of therapeutics	The article reviews the role of mifepristone in CS treatment	Mifepristone is recommended for CS to control hyperglycemia and those who are not candidates for surgical treatment. The recommended starting dosage is 300 mg/day. Maximum dosage is 1,200 mg/day
	Fleseriu et al. [69]	Clinical trial	50 adults with endogenous CS and associated type 2 diabetes mellitus/impaired glucose tolerance (C-DM) or a diagnosis of hypertension (C-HT)	In C-DM: Significant decrease in HbA1c, decrease in fasting plasma glucose. In C-HT: Improvement in DBP, decreased waist circumference, and mean weight change
	Katznelson et al. [70]	Clinical trial	46 adult patients with refractory CS along with type 2 DM or impaired glucose tolerance, and/or a diagnosis of hypertension	88% of patients had clinically significant improvement at 24 weeks
Insulin sensitivity and resistance	Gubbi et al. [72]	Randomized controlled trial	16 overweight individuals with pre-diabetes or mild type 2 DM but not clinical hypercortisolism studied	Short-term mifepristone administration improves adipose and hepatic insulin sensitivity among obese individuals with hyperglycemia without hypercortisolism by GR blockade
Cancer	Nanda et al. [73]	Randomized controlled trial	Nine patients were enrolled. Patients were randomized to placebo or mifepristone for the first cycle; mifepristone was given to all for subsequent cycles	Immunohistochemical staining for GR found six of nine tumors were GR-positive. All six GR-positive tumors were triple-negative at the time of recurrence. Of these six patients, two had a complete response, two had partial responses, one had stable disease, and one had progressive disease. GR inhibition by mifepristone plus chemotherapy produces manageable toxicity
	Liu et al. [74]	An experimental study (cell line study)	A compound library containing 17 compounds by altering the sensitive metabolic region of mifepristone is studied for the treatment of TNBC cell lines	FZU-00,003 displayed the most potent efficiency
	Kim et al. [75]	An experimental study (mouse model)	Mouse model of ovarian cancer that mimics the clinical metastases of human high-grade serous carcinoma	An effective chemopreventive strategy in the BRCA gene mutation for ovarian cancer and by extension breast cancer
	Goyeneche et al. [76]	An experimental study (cell line and animal study)	Ovarian cancer cell lines of different genetic backgrounds (SK-OV-3, Caov-3, OV2008, and IGROV-1)	In vitro, mifepristone inhibited ovarian cancer cell proliferation in a dose- and time-dependent manner. In vivo, mifepristone significantly delayed the growth of ovarian carcinoma xenografts in a dose-dependent manner
	Serritella et al. [77]	Clinical trial	106 patients with castration-resistant prostate cancer	Enzalutamide combined with mifepristone was safe and well-tolerated but did not meet its endpoint
Meningioma	Ji et al. [81]	Randomized controlled trial	164 eligible patients, 80 were randomly assigned to mifepristone and 84 to placebo	Twenty-four patients (30%) were able to complete 2 years of mifepristone without disease progression. There was no statistical difference in failure-free or overall survival
	Llaguno-Munive et al. [83]	An experimental trial (animal study)	Wistar rats were studied after orthotopically implanting C6 glioma cells	Mifepristone could act as a chemosensitizing agent for temozolomide during chemotherapy for GBM
Ocular pathology				
Uveal melanoma	Alvarez et al. [84]	An experimental study (cell lines)	In vitro, uveal melanoma cells were incubated with mifepristone for up 72 hours	Mifepristone inhibits functionality, growth capacity, and viability of human primary and metastatic uveal melanoma cell lines in a concentration-related manner
Glaucoma	Green et al. [86]	An experimental study (animal study)	Topically administered mifepristone	Mifepristone alone reduced IOP relative to controls and the further addition of medrysone (progesterone) at 14 days did not affect IOP. Mifepristone is an effective antagonist against progesterone’s effects on IOP
	Green et al. [87]	An experimental study (animal study)	Topical dexamethasone interaction with mifepristone	Mifepristone caused a lower intraocular pressure than seen in other groups, whether in the presence or absence of dexamethasone
Psychiatric diseases				
Depression	Flores et al. [88]	Randomized controlled trial	30 patients’ data were analyzed. 15 were randomized to placebo, and 15 were randomized to mifepristone for 8 days	Short-term use of mifepristone may be effective in the treatment of psychotic major depression and may re-regulate the HPA axis
Alcohol dependence and withdrawal	Khom et al. [89]	An experimental study (animal study)	67 adult, male Sprague-Dawley rats with alcohol vapor dependence	Mifepristone decreased GABA release with the largest effect in dependent rats
Olanzapine-induced weight gain	Beebe et al. [90]	An experimental study (animal study)	Adult female Sprague-Dawley rats grouped to olanzapine and mifepristone	Results suggest that mifepristone, a potent glucocorticoid antagonist, may both reduce and prevent olanzapine-induced weight gain in rats
Alzheimer’s disease (AD)	Belanoff et al. [93]	Clinical trial	The rate of cognitive decline is compared in AD subjects randomized to receive 200 mg of mifepristone daily for 6 months or a placebo	Mifepristone decelerated the rate of cortisol-related cognitive decline in subjects with mild-to-moderate AD

## Conclusions

At present, the most popular use of mifepristone is for medical abortion because of its effectiveness and safety. Its effective use in other indications in obstetrics, gynecology, oncology, and endocrinology needs further evaluation, although the present study has shown some encouraging results which would bring increased access to various dosage formulations and options for healthcare globally.
